# Angiopoietin-like Proteins and Lipoprotein Lipase: The Waltz Partners That Govern Triglyceride-Rich Lipoprotein Metabolism? Impact on Atherogenesis, Dietary Interventions, and Emerging Therapies

**DOI:** 10.3390/jcm13175229

**Published:** 2024-09-04

**Authors:** Alejandro Gugliucci

**Affiliations:** Glycation, Oxidation and Disease Laboratory, Touro University California, Vallejo, CA 94592, USA; alejandro.gugliucci@gmail.com

**Keywords:** LPL, TRL, ANGPTL, apoCIII, chylomicrons, VLDL, LDL, atherogenesis, remnants, apoB100, apoB48

## Abstract

Over 50% of patients who take statins are still at risk of developing atherosclerotic cardiovascular disease (ASCVD) and do not achieve their goal LDL-C levels. This residual risk is largely dependent on triglyceride-rich lipoproteins (TRL) and their remnants. In essence, remnant cholesterol-rich chylomicron (CM) and very-low-density lipoprotein (VLDL) particles play a role in atherogenesis. These remnants increase when lipoprotein lipase (LPL) activity is inhibited. ApoCIII has been thoroughly studied as a chief inhibitor and therapeutic options to curb its effect are available. On top of apoCIII regulation of LPL activity, there is a more precise control of LPL in various tissues, which makes it easier to physiologically divide the TRL burden according to the body’s requirements. In general, oxidative tissues such as skeletal and cardiac muscle preferentially take up lipids during fasting. Conversely, LPL activity in adipocytes increases significantly after feeding, while its activity in oxidative tissues decreases concurrently. This perspective addresses the recent improvements in our understanding of circadian LPL regulations and their therapeutic implications. Three major tissue-specific lipolysis regulators have been identified: ANGPTL3, ANGPTL4, and ANGPTL8. Briefly, during the postprandial phase, liver ANGPTL8 acts on ANGPTL3 (which is released continuously from the liver) to inhibit LPL in the heart and muscle through an endocrine mechanism. On the other hand, when fasting, ANGPTL4, which is released by adipocytes, inhibits lipoprotein lipase in adipose tissue in a paracrine manner. ANGPTL3 inhibitors may play a therapeutic role in the treatment of hypertriglyceridemia. Several approaches are under development. We look forward to future studies to clarify (a) the nature of hormonal and nutritional factors that determine ANGPTL3, 4, and 8 activities, along with what long-term impacts may be expected if their regulation is impaired pharmacologically; (b) the understanding of the quantitative hierarchy and interaction of the regulatory actions of apoCIII, apoAV, and ANGPTL on LPL activity; (c) strategies for the safe and proper treatment of postprandial lipemia; and (d) the effect of fructose restriction on ANGPTL3, ANGPTL4, and ANGPTL8.

## 1. Introduction

The concept of LDL-C-driven atherogenesis has been known as the “LDL-hypothesis” for many years [1,2,3,4]. Nevertheless, it is no longer appropriate to treat this as a hypothesis or to concentrate only on LDL-C (the LDL-centric paradigm) due to the overwhelming evidence supporting the role of other ApoB100 and apoB48-containing lipoproteins in atherogenesis [1,5,6,7,8,9,10,11,12]. The current understanding that these lipoproteins are causative in atherosclerotic cardiovascular disease (ASCVD) and that reducing their level is the cornerstone of prevention may be better conveyed by the term “ApoB principle” according to some authors [1]. This represents conceptual progress, but it needs deeper insight as it does not specify which apoB they are referring to. After successful statin medication, there is a 50% residual risk for arteriosclerotic cardiovascular disease due to other lipid abnormalities and immunological issues [3,5,13,14,15]. Over the past ten years, triglyceride-rich lipoproteins (TRL) and their hydrolysis products, called “remnants”, have thus gained significance in the context of atherogenesis. The following are key facts to consider:

Serum triglycerides (TG) are inherently healthy and providers of most of our circulating fuel. Indeed, TG are in fact inert markers of remnant TRL that usually contain much more cholesterol than low-density lipoproteins (LDL) and are possibly even more atherogenic [7,9]. 

The main cause of the accumulation of these partially lipolyzed derivatives is the inadequate catabolism of TRL. The so-called “ApoB principle” has been supported by strong and consistent evidence from basic science and animal research, epidemiologic cohorts, genetic analyses (including MR), and RCTs. These studies have demonstrated that ApoB100 particles initiate and propagate atherosclerosis and satisfy all requirements for a causal role in atherogenesis and ASCVD [16,17,18,19]. 

Not only apoB100 but apoB48 as well as chylomicrons (CM) should not be neglected since we spend most of our day in the postprandial phase [6,20,21]. It is now widely known that all apoB100 lipoproteins, including TG-rich particles such as remnants and VLDL as well as Lp [a], may cross the endothelial border [17]. Somewhat neglected, though, is the fact that a large proportion of those TRL are chylomicrons (CM) that do not contain apoB100, but apoB48 [6,9,21,22]. Indeed, the “advanced lipid panel” employed in the clinic merely reports “apoB” concentrations, without specifying whether it is apoB100, apoB48, or both. Retention of these particles (apoB48 and apoB100-containing remnants) in the vascular intima sets off a series of intricate events that culminate in the formation of atherosclerotic plaque. These events include the formation of foam cells, the release of proinflammatory mediators, the deposition of extracellular lipid deposits and cholesterol crystals, and the activation of innate and adaptive immune responses. 

At the center of the whole orchestration of TRL catabolism lies lipoprotein lipase (LPL). Its mechanism and regulation have been the focus of intense research which flourished in the past few years to the point of producing several targets for therapeutics, as we shall see, some of which are reaching the market [23,24,25]. In essence, remnant cholesterol-rich **chylomicrons** and VLDL particles play a role in atherogenesis. These remnants increase when lipoprotein lipase (LPL) activity is inhibited. ApoCIII has been thoroughly studied in this regard and therapeutic options to curb its effect are available [25]. 

In addition to apoCIII regulation of LPL activity, there is a more precise control of LPL in various tissues, which makes it easier to physiologically divide the TRL burden according to the body’s requirements. In general, oxidative tissues such as skeletal and cardiac muscle preferentially take up lipids during fasting, whereas adipocyte storage is not preferred. It is now clear that LPL activity cross-regulation between muscle and fat is essential to the trafficking and partitioning of TG. 

Aims of this perspective. Given the foregoing, this perspective addresses the recent improvements in our understanding of circadian LPL regulations and their therapeutic implications. Three major tissue-specific lipolysis regulators were identified: ANGPTL3, ANGPTL4, and ANGPTL8. In summary during the postprandial phase, liver ANGPTL8 acts on ANGPTL3 (which it releases continuously) to inhibit LPL in the heart and muscle through an endocrine mechanism. On the other hand, when fasting, ANGPTL4, which is released by adipocytes, inhibits lipoprotein lipase in adipose tissue in a paracrine manner [7,26]. By virtue of ANGPTL3’s crucial role in this process and the findings of loss of function studies in humans and animals, ANGPTL3 inhibitors may play a therapeutic role in the treatment of hypertriglyceridemia. 

Let us then first succinctly review some major concepts on TRL metabolism and then bring to the forefront the most recent data on ANGPTL3, ANGPTL4, and ANGPTL8 biology, therapeutic implications, and suggest potential research topics that may pave the way for future advances in the subject.

## 2. Lipoprotein Lipase Structure and Function

**Figure 1:**
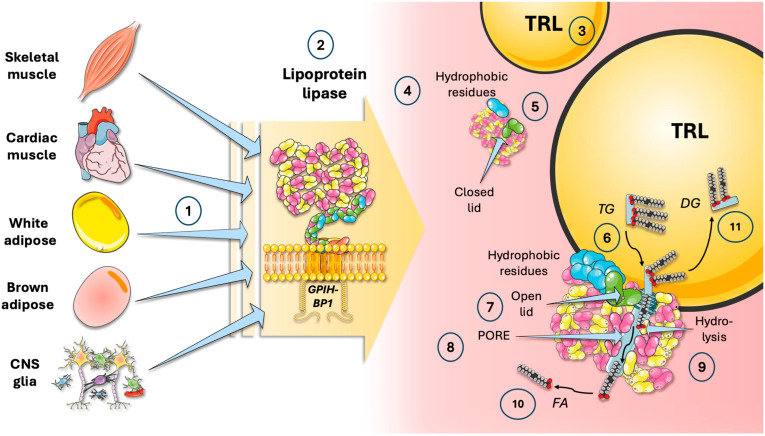
**Lipoprotein lipase (LPL) is the key enzyme that transforms insoluble, energy-rich triglycerides (TG) into soluble fatty acids (FA); some structural considerations are as follows.** (1) Skeletal and cardiac muscle, adipocytes (white and brown), oligodendrocytes, astrocytes, and microglia, among others, produce lipoprotein lipase (2). Anchored in the adjacent endothelium by glycosylphosphatidylinositol-anchored high-density lipoprotein binding protein 1 (GPIHBP1), it acts on triglyceride-rich lipoproteins (TRL) 3) LPL binds to TRL via (4) hydrophobic residues, its hydrolytic pore closed (5) until the binding occurs. LPL acts on TG (6) when ensuing conformational changes result in (7) opening of the lid of the pore (8) where hydrolysis occurs (9), and in a unidirectional flow, one FA at a time (10) is released, as well as the resulting di- and mono-glycerides (11). The figure was partly generated using Servier Medical Art, provided by Servier, licensed under a Creative Commons Attribution 3.0 unported license.

LPL is a complex enzyme expressed in oxidative cells (skeletal and cardiac myocytes, macrophages, glia) and adipose cells (both white and brown), among others (Figure 1). It anchors in the adjacent endothelium by glycosylphosphatidylinositol-anchored high-density lipoprotein binding protein 1 (GPIHBP1) and acts on TRL [27]. LPL binds to TRL via hydrophobic residues. LPL acts on these TG catalyzing their hydrolysis in a unidirectional flow one FA at a time is released, together with the resulting glycerol [3,6,7,23,28,29,30,31].

**Figure 2:**
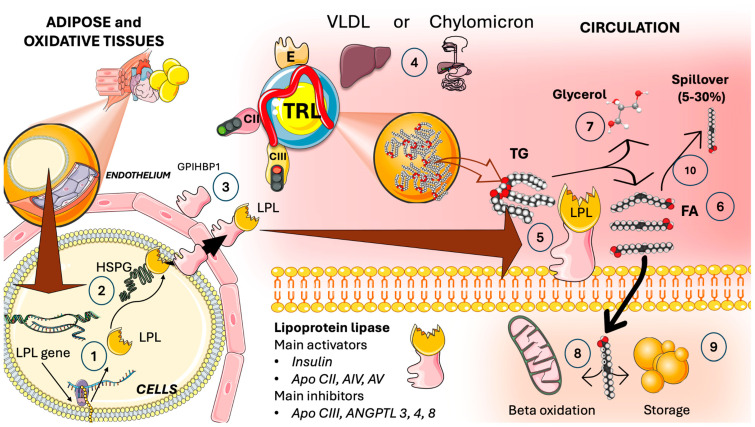
**Capillary lipoprotein lipase (LPL) directs the flux and partition of fatty acid supply to tissues: main features.** (1) Adipocytes, and cells in oxidative tissues such as skeletal and cardiac muscle, among others, produce lipoprotein lipase. (2) LPL is a complex enzyme that needs heparin sulfate proteoglycans (HSPG) on the cell surface to aid in the molecule’s transcytosis to the microvascular’ luminal surface. (3) Glycosylphosphatidylinositol-anchored high-density lipoprotein binding protein 1 (GPIHBP1) assists in fixing LPL on the luminal surface of the endothelial cells and supports the configuration of LPL to an active lipolytic enzyme. (4) LPL acts on triglyceride-rich lipoproteins (TRL), namely VLDL (all day) and chylomicrons (postprandial), to hydrolyze triglycerides (TG) (5) into free fatty acids (FA) (6) and glycerol (7). Free fatty acids are employed for oxidation in muscle and myocardial tissue as shown in (8) or storage in adipocytes as shown in (9). (10) About 5 to 30% may remain in the circulation, referred to as “spill over” fatty acids. LPL has a very multifaceted regulation: its chief activators are insulin, apoCII, AIV, and AV, and its key inhibitors are apoCIII and angiopoietin-like proteins (ANGPTL) 3, 4, and 8. The figure was partly generated using Servier Medical Art, provided by Servier, licensed under a Creative Commons Attribution 3.0 unported license.

In Figure 2, we illustrate that LPL secretion and adequate positioning in the luminal membrane of the endothelia is a complex process that requires heparan sulfate proteoglycans (HSPG) on the cell surface to aid in the molecule’s transcytosis to this microvascular’ luminal surface. There, LPL is transferred to GPIHBP1 which also supports the configuration of LPL to an active lipolytic enzyme [23,24,30,32]. Previously, it was believed that LPL only functioned as a dimer; it recently became clear that the monomer bound to GPIHBP1 is very active [23,32]. LPL acts on VLDL (all day) and chylomicrons (postprandial) to hydrolyze TG into free fatty acids (FA) and glycerol. Free fatty acids are employed for oxidation in muscle and myocardial tissue or storage in adipocytes, as shown in the figure. More than 5% may remain in the circulation, referred to as “spill over” FA, which is re-esterified later in the liver. 

LPL has a multifaceted regulation: its chief activators are insulin, apoCII, AIV, and AV, and its key inhibitors are apoCIII and angiopoietin-like proteins (ANGPTL) 3, 4, and 8. The latter group is the focus of this article [23,28,31,33,34,35,36]

Of note, LPL’s function appears to be fine-tuned by inhibition. LPL is a high-capacity, very effective enzyme for removing TG from the center of TRL particles. In our evolutionary history, the availability of fatty meals was sporadic and required a continuous energy expenditure. This was particularly true of food derived from animals. Baseline slowing of TRL catabolism by inhibition of LPL probably functions to ensure distribution to multiple tissue sites rather than having the entire TG load in a chylomicron or VLDL particle removed at “first pass”, depriving the rest of the body of much-needed sustenance [9,37,38]. It appears that to maintain a circulating mass of TG available before the next meal and for as long as feasible, nature favored or preferred a constant state of inhibition of LPL. It must be recalled that skeletal, and more so, cardiac myocytes “prefer” FA as the main substrate for oxidation, considering the much higher yield of ATP per mole (36 moles per mole of glucose versus over 120 moles per FA mole on average). 

LPL inhibition is conducted by apoCIII, and ANGPTL3, 4, and 8. It is easy to infer that this beneficial effect when food is either scarce or of uncertain periodicity becomes a liability in our time of excess ingestion of lipids and carbohydrates and the fact that we spend most of the day in the postprandial phase with excess chylomicrons in circulation. Therein lies the significance of the residual risk even assuming optimal LDL-C values [3,5,13,39]. 

## 3. Overview of Main LPL Regulators

The quick adaptive alterations of LPL to dietary changes are controlled by insulin responses in conjunction with the moderating effects of other hormones and proteins that regulate necessary adjustments in the lipolytic rates between fasting and postprandial states. Over an appropriate endocrine milieu where insulin plays a master role, some *apolipoproteins*, angiopoietin-like proteins (ANGPTLs, see specifics in Section 5), and recently discovered factors exert the fine regulation.

### 3.1. Apolipoproteins (Apo)

Many apolipoproteins on the surface of TRL have different effects on their metabolism. As opposed to the structural apoB100 and 48, apoCI, apoCII, apoCIII, and apoE are surface proteins exchanged between TRL and HDL particles depending on the nutritional or metabolic state. 

APOCII promotes the hydrolysis of TG into FA by activating LPL. Individuals with APOCII deficiency have severe hypertriglyceridemia and poor activation of LPL. ApoCII seems to be a rate-limiting factor in LPL-mediated lipolysis. Based on these data, a dual apoCII mimetic-apoCIII antagonist peptide was found to be effective in reducing plasma triglyceride levels. The initial stages of development for these agents are currently ongoing [23,24,29,31].

APOCIII’s main function is the modulation of lipid metabolism to control blood TG levels. APOCIII inhibits the clearance of TRL particles and adversely influences LPL function. Because APOCIII promotes the buildup of serum TG, high levels of this protein are linked to an increased risk of hypertriglyceridemia and cardiovascular disease (CVD). Phase II and phase III clinical trials are currently testing antisense oligonucleotides that target APOCIII mRNA (Volanesoren and Olezarsen). In both animal and human investigations, these drugs reduce the plasma levels of apoCIII by 70–80% [9,15,25,37,38,40].

Apolipoprotein AV. A significant function of ApoAV is to regulate plasma TG levels and lipid metabolism. Primarily produced in the liver, ApoAV interacts with and influences the metabolism of chylomicrons and VLDL. By boosting the degradation of TRL, promoting their removal from the circulation, and positively controlling LPL activity, it controls TG levels [41,42,43] mainly by inhibiting the ANGPTL3/8 complex [44,45], as we elaborate on later.

### 3.2. Angiopoietin-like Proteins (ANGPTLs)

ANGPTL3, 4, and 8 are the latest discoveries in LPL regulation and have a significant influence on LPL activity by coordinating the needs of oxidative tissues and fat in the fast and fed circadian cycles, as we detail further in this article. This exciting field has flourished and delivered new therapeutic targets currently under intense scrutiny. 

### 3.3. CREBH

The transcription factor CREBH (cAMP response element-binding protein, hepatocyte-specific), which is predominantly present in the liver, controls lipid metabolism. Many lipid metabolism-related genes, including those involved in TG synthesis and transport, are upregulated in response to CREBH activation. The activation of CREBH facilitates TG lipolysis and fatty acid oxidation in situations of metabolic stress or fasting [46]. Very recently, research has shown that CREBH can cleave to release its C-terminal portion (CREBH-C), which is secreted into the bloodstream and functions as a hepatokine to control LPL activity, as we further describe later in this article [46,47].

## 4. The Need for Tissue-Specific Regulation of LPL in the Fast-Fed Cycle

As we have stated, LPL serves as a gatekeeper for tissue-specific fat transport, allowing for either storage or usage to continuously optimize the exchange of TG within the organism. Its function varies according to the body’s state of feeding or fasting. TG can be taken up by WAT because postprandial LPL activity rises in WAT but decreases in oxidative tissues. In contrast, oxidative tissues experience an increase in fasting LPL activity, ensuring that TGs are absorbed by these tissues to fulfill the body’s energy requirements. 

Before analyzing the factors that govern this careful cross-regulation, let us conduct a thought experiment and imagine what would occur during fasting (for example) if that regulation did not exist. 

**Figure 3:**
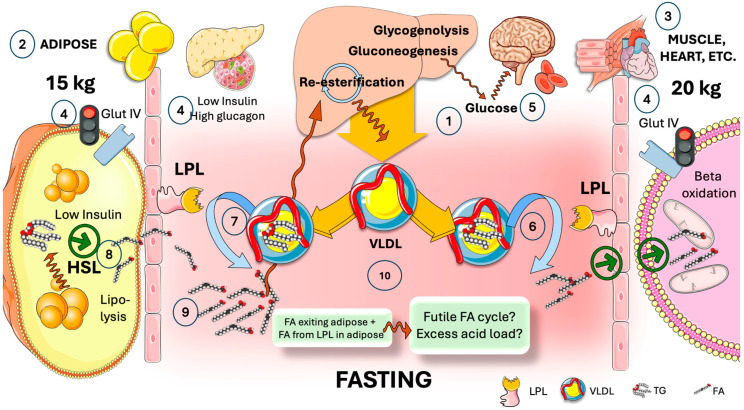
**Potential waste of energy-rich fatty acids during fasting if LPL were not tissue-specifically regulated. Competition between adipose and oxidative tissues in the fasting state.** (1) During fasting, the liver produces VLDL as a source of fatty acids (FA) for most oxidative tissues. (2) VLDL circulates and reaches both adipose and (3) oxidative tissues. (4) Low insulin and high glucagon promote hepatic production of glucose whereas GlutIV is inactive, and (5) most glucose is therefore taken up by the brain and erythrocytes via GlutI and II. As shown in Figure 1, VLDL reaches LPL (6) in oxidative tissues, providing most of the energy as FA. At the same time, VLDL also reaches LPL (7) in adipose, where hormone-sensitive lipase (HSL) fully activated by low insulin catalyzes extensive lipolysis (8), inundating the capillary with intracellular FA that would add to the extracellular FA produced by LPL (9). Given the relative masses of muscle and adipose (rough averages are presented), between 30 and 40% of very energy-demanding productions of TG by the liver, exported as VLDL, would possibly enter a liver-adipose-liver futile FA cycle, which would also add to the acid load of the bloodstream (10). This is prevented by the presence of another level of regulation provided by the axis of ANGPTL3, 4, and 8 that controls the partition of TRL fluxes during fasting and feeding cycles, as depicted in Figure 3 and Figure 4. The Figure was partly generated using Servier Medical Art, provided by Servier, licensed under a Creative Commons Attribution 3.0 unported license.

In Figure 3, we show there is a potential waste of energy-rich FA during fasting if LPL were not tissue-specific regulated. During fasting, the liver produces VLDL as a source of FA for most oxidative tissues. VLDL circulates and reaches both adipose and oxidative tissues (very roughly 15 kg vs. 20 kg of tissue mass in an average 70 kg person). Low insulin and high glucagon during fasting promote hepatic production of glucose; most glucose is therefore taken up by the brain and erythrocytes via GlutI and II because Glut IV is inactive in adipose and muscle; VLDL reaches LPL in oxidative tissues, providing neutral TG that is hydrolyzed by it into FA which supplies most of the skeletal and cardiac myocyte metabolic needs in this state [7]. At the same time, VLDL also reaches LPL in adipose, where hormone-sensitive lipase (HSL) is fully activated by the presence of low insulin levels. HSL catalyzes extensive lipolysis, thereby flooding the capillary with intracellular FA that would add to the extracellular FA produced by LPL. Considering the proportional masses of muscle and adipose tissue (rough averages are shown), between 30 and 40 percent of the liver’s extremely energy-demanding TG synthesis, exported as VLDL, may potentially enter a liver–adipose–liver futile FA cycle, which would also raise the blood’s acidity, putting pressure on buffer systems and the kidney. 

This is thwarted by the existence of an additional regulatory layer supplied by the axis ANGPTL3, 4, and 8, which governs the division of TRL fluxes during cycles of fasting and feeding. In the subsequent sections, we provide a detailed explanation.

## 5. Adipocyte ANGPTL4 Diverts Most TRL during Fasting to Oxidative Tissues by Transiently Blocking Adipose Tissue LPL Activity

**Figure 4:**
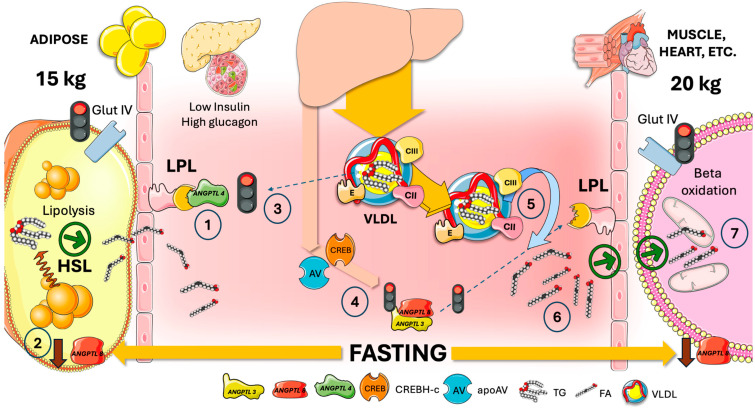
**ANGPTL4 acts in an autocrine and paracrine fashion in adipose to divert VLDL to oxidative tissues during fasting.** Superimposed on the regulation of LPL activity by apoCII/CIII, there exists a finer control of the enzyme in different tissues that provides for the physiological partition of the TRL load depending on the needs of the body. Basically, during fasting, lipids are preferentially taken up by oxidative tissues such as cardiac and skeletal muscle, and storage at the adipocytes is not favored. Thus, during fasting, the futile cycle depicted in Figure 2 is prevented (1) by ANGPTL4, which is produced by adipocytes during fasting, whereas ANGPTL8 is downregulated (2). ANGPTL8 curbs ANGPTL4 inhibition of LPL. The resulting mainly inactive LPL (3) shunts VLDL away from adipose. Additionally, fasting increases the hepatic output of apoAV and CREBH (cAMP response element-binding protein, hepatocyte-specific (4), which inactivates the ANGPTL3/8 complex, a potent inhibitor of oxidative tissue LPL, which is key in the fed state (see Figure 4). The combined effect allows for 5) an unrestricted activity of oxidative tissue LPL (regulated, however, by the apoCII/CIII balance) providing much-needed FA (6) for energy production in glucose-depleted muscle (7). The Figure was partly generated using Servier Medical Art, provided by Servier, licensed under a Creative Commons Attribution 3.0 unported license.

ANGPTL4 is a member of the large ANGPTL family (1–10 are described). It is activated by fasting through adipocytes’ peroxisome proliferator-activated receptor (PPAR). By means of its regulation of LPL, ANGPTL4 plays a crucial role in the regulation of fat metabolism [48,49,50,51]. As shown in Figure 4, being a strong LPL inhibitor, ANGPTL4 is decisive in controlling LPL activity when fasting. Neutralizing antibody-treated wild-type (WT) and ANGPTL4 knockout (KO) mice consistently exhibit lower plasma TG levels and higher post-heparin plasma LPL activity [26,47]. Although ANGPTL4 is also expressed in both the liver and adipose, it primarily controls TG partitioning in the white adipose tissue (WAT) but not in the liver. Additional roles of ANGPTL4 in the integrity of the intestinal barrier and interaction with microbiota are under intense scrutiny [49,51,52,53,54]. WAT ANGPTL4 regulates LPL in both an autocrine and paracrine fashion during fasting by suppressing LPL activity. ANGPTL4 has been shown to inhibit LPL through a variety of mechanisms. It facilitates the unfolding of LPL, which in turn causes LPL to be cleaved and degraded [55]. LPL’s affinity for binding to GPIHBP1 can likewise be decreased by ANGPTL4 [26,47].

As a result, targeting ANGPTL4 has emerged as a prospective therapeutic strategy for the treatment of dyslipidemia and CVD. Figure 4 also illustrates the role of ANGPTL8 (see more detail below) in this process. Fasting reduces ANGPTL8 expression. When ANGPTL8 binds to ANGPTL4, the complex loses its inhibitory capacity. Low ANGPTL8 then results in full inhibition of WAT LPL. VLDL is thus diverted away from adipose tissue [47]. 

**Figure 5:**
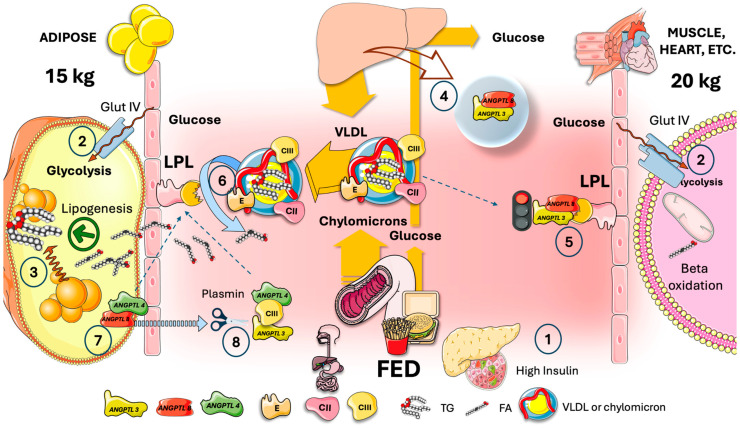
**Hepatic ANGPTL3 and 8 act in an endocrine fashion on muscle to divert VLDL and Chylomicrons to adipose tissue in the fed state.** Superimposed on the regulation of LPL activity by apoCII/CIII, there exists a finer control of the enzyme in different tissues that provides for the physiological partition of the TRL load depending on the needs of the body. Upon feeding LPL, activity in adipocytes is much higher, and at the same time, its activity is reduced in oxidative tissues. In a nutshell, ANGPTL3 (secreted all day long) from the liver acts in an endocrine way to inhibit lipoprotein lipase in muscle and heart during the postprandial period. During the fed or postprandial phase, high insulin levels (1) promote the uptake of glucose via GlutIV activation in adipose and muscle (2) as well as lipogenesis in the former (3). Feeding induces high expression of ANGPTL8 (4), which multiplies ANGPTL3 inhibition of LPL several-fold (5). TRL (VLDL and chylomicrons) are then preferentially acted upon on adipose (6). Feeding enhances ANGPTL8 synthesis and reduces ANGPTL4 production in adipose, which then liberates adipose LPL (7). Moreover, plasminogen is attracted to ANGPTL4/8 together with tPA, which turns plasminogen into plasmin. Without influencing the LPL activator APOCII, plasmin then cleaves LPL inhibitors such as ANGPTL3/8, A4, and APOCIII (8). Through this sequence of events, LPL activity is entirely restored locally in adipose, whereas ANGPTL3/8 can precisely block oxidative-tissue LPL (5). One should take note of the competition between intestinal chylomicrons and hepatic VLDL for hydrolysis by LPL, which happens mostly in the capillaries of adipose tissue. Fat is thus preferentially partitioned to adipose tissue for storage following a meal. Note that this regulation is a fine-tuning of the control provided by insulin and the rate of apoCII and apoCIII on TRL. We go into additional detail in this article regarding the potential significance of ANGPTL3 inhibitors as a treatment route for hypertriglyceridemia, given the critical role ANGPTL3 plays in this process and the findings of loss of function studies conducted on humans and animals. The figure was partly generated using Servier Medical Art, provided by Servier, licensed under a Creative Commons Attribution 3.0 unported license.

Moreover, as also depicted in Figure 4, fasting raises the amounts of apoAV and CREBH that are produced in the liver. These proteins inactivate the remaining ANGPTL3/8 complex in the circulation since the last meal, which is a powerful inhibitor of oxidative tissue LPL that is essential in the fed state (see Figure 5). The combined result permits the unlimited activity of oxidative tissue LPL (controlled by the apoCII/CIII balance, however), which supplies much-needed FA for the generation of energy in muscle that is glucose-depleted during fasting [7,47]. 

## 6. In the Fed State, Hepatic ANGPTL3/8 Acts on Muscle in an Endocrine Manner to Redirect VLDL and Chylomicrons to Fat Tissue

This control, depicted in Figure 5, enables the physiological partitioning of the TRL burden based on the body’s needs. LPL activity in adipocytes increases significantly after feeding, while in oxidative tissues, it decreases concurrently.

ANGPTL3 is mostly generated and secreted by the liver and is important in regulating blood levels of TG and cholesterol [48,56,57]. ANGPTL3 works by preventing the hydrolysis of TG and high-density lipoproteins (HDL) in peripheral tissues by endothelial lipase (EL) and LPL, respectively. ANGPTL3 raises circulating lipoproteins such as chylomicrons and VLDL (and consequently their remnants) by decreasing the hydrolysis of TG from these lipoproteins ymes. The intimate mechanisms of action of ANGPTL3 are less well described than those of ANGPTL4 [6,33,36]. 

As shown in Figure 5, during the postprandial phase the liver’s ANGPTL3 (which is released continuously) inhibits lipoprotein lipase in the heart and muscle through an endocrine mechanism. In the postprandial phase, high insulin/low glucagon levels facilitate glucose uptake via GlutIV transfer to membranes in adipose and muscle as well as lipogenesis in the former. Feeding causes ANGPTL8 to be highly expressed, multiplying ANGPTL3 suppression of LPL several times by forming 3/1 ANGPTL3/8 complexes [9,15,21,47]. TRL (VLDL and chylomicrons) are thus shunted to adipose tissue [58]. Feeding also increases the synthesis of ANGPTL8 and decreases the generation of ANGPTL4 in adipose tissue, hence removing LPL inhibition. More recently, a supplementary role was discovered for tPA and plasminogen in this process [47,59]. Plasminogen and tPA bind to the ANGPTL4/8 complex whereupon plasminogen is converted to plasmin [59]. Plasmin then cleaves LPL inhibitors including A3/8, A4, and APOCIII, without affecting the LPL activator APOCII [47,59,60]. This series of events restores LPL activity completely locally in adipose tissue, while ANGPTL3/8 can precisely block LPL in oxidative tissues. It is important to consider the competition that exists between intestinal chylomicrons and hepatic VLDL for the hydrolysis of LPL, which primarily occurs in adipose tissue capillaries. This explains the rise in VLDL TG in the postprandial phase. In summary, after a meal, fat is preferentially partitioned to adipose tissue for storage [6,7,20,21,61,62]. Be aware that the rate of apoCII and apoCIII on TRL as well as the insulin-provided control are being fine-tuned by this regulation. 

Given the crucial role ANGPTL3 plays in this process and the results of loss of function studies conducted on hypertriglyceridemia, inhibiting ANGPTL3 activity may provide a pharmacological approach for treating dyslipidemia and lowering the risk of CVD, according to research and clinical investigations on the protein. Section 11 provides a more comprehensive examination of the potential therapeutic impact of ANGPTL3 inhibitors.

## 7. ANGPTL8, the Main Circadian LPL Switch? 

Before summarizing the major components of the fast and fed regulation of LPL, it is important to briefly review recent developments regarding the increasingly critical role that ANGPTL8 has as a prominent actor in the process.

A decade ago, many researchers reported the functional roles of a hitherto uncharacterized gene for lipid metabolism under several names, including lipasin, which was subsequently renamed ANGPTL8 [33,35,36,63,64].

Food consumption strongly induces the expression of ANGPTL8, which is exclusive to the liver and fat as identified by pioneer work in Hobbs’s laboratory [58,65,66,67]. ANGPTL8 is also widely present in brown fat, where exposure to cold stimulates its expression. Recent evidence clearly demonstrated that circulating ANGPTL8 originates primarily from hepatic secretion rather than adipocytes [35,68]. The LPL inhibitory activity of ANGPTL8 is due to the formation of a compound with ANGPTL3 [65,69,70]. After being secreted into the bloodstream, liver ANGPTL8 produces the ANGPTL3/8 complex (ratio 3:1), which suppresses oxidative-tissue LPL via an endocrine mechanism [67]. Moreover, as adipocytes ANGPTL8 and ANGPTL4 combine to generate a complex that reverses ANGPTL4’s inhibition of LPL [47,63]. Two negatives make a positive, and ergo ANGPTL8 stimulates adipose LPL activity [66]. In mice, ANGPTL8 inhibition with a monoclonal antibody increases TG clearance and produces weight loss; in dyslipidemic cynomolgus monkeys, this antibody normalizes plasma TG levels [47]. Moreover, an antibody specific to the ANGPTL3/8 complex significantly reduces TG levels in mice and humans and prevents ANGPTL3/8 inhibition of LPL [35].

Human serum levels of ANGPTL8 have been shown to physiologically drop during an overnight fast and rise two hours after a specified meal. Circulating levels of ANGPTL8, ANGPTL3/8, and ANGPTL4 /8 are increased by food intake and are lowered by fasting or exercise. A wealth of data from GWAS in humans and loss- and gain-of-function studies in mice have shown that ANGPTL8 is a feeding-induced hepatokine that regulates LPL activity by building complexes with ANGPTL3 and ANGPTL4. The correlation between ANGPTL8 levels and various clinical disorders, as well as its non-metabolic functions, including inflammation, has been explored [35].

## 8. Cross-Regulation of LPL in the Fast-Fed Cycle, Current Model

**Figure 6:**
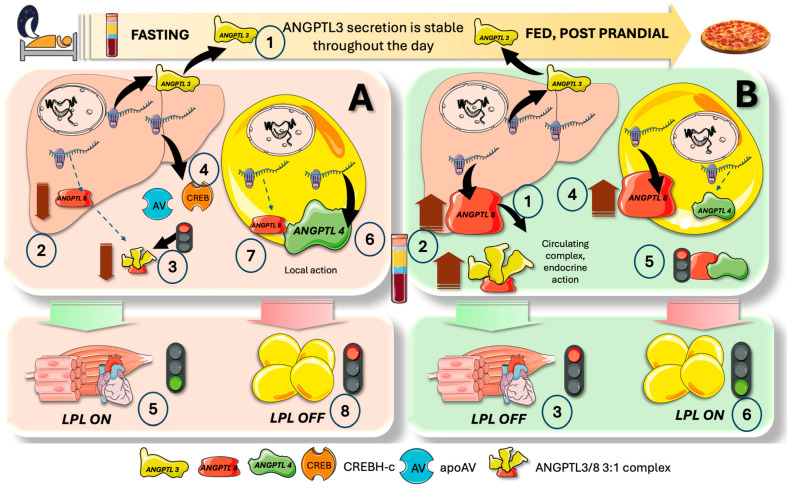
**Integrated current view of the ANGPTL3,4,8 axis on fine-tuning of LPL activity and fat partition in the fast-fed cycle**. (1) The liver produces and secretes relatively steady levels of ANGPTL3. In the fasting state (**left** column, A), ANGPTL8 is reduced (2), and therefore the levels of the active LPL inhibitor which is a 3/1 ANGPTL3/8 complex are greatly reduced (3). Additionally, fasting increases the hepatic output of apoAV and CREBH (cAMP response element-binding protein, hepatocyte-specific) (4), which inactivates the ANGPTL3/8 complex. Therefore, all inhibition of muscle LPL is lifted, and oxidative tissues receive FA from VLDL (5). In parallel, (6) and (7) fasting greatly increases the production of ANGPTL4 (and lowers ANGPTL8) whereby ANGPTL4 locally blocks adipose LPL (8), preventing VLDL hydrolysis and shunting VLDL to oxidative tissues. Potential futile cycles are prevented. In the fed state (**right** column, B), hepatic ANGPTL8 is greatly increased (1), and therefore the levels of the active LPL inhibitor, which is a 3/1 ANGPTL3/8 complex, are increased (2), acting as endocrinal mediators on LPL in oxidative tissues to block it (3). Additionally, feeding increases ANGPTL8 expression in adipose while reducing ANGPTL4 (4). The ANGPTL4/8 complex does not inhibit LPL (5) Therefore, all inhibition of adipose LPL is lifted (5), and adipocytes can receive FA from VLDL and chylomicrons for appropriate storage stimulated by high insulin levels (6). The figure was partly generated using Servier Medical Art, provided by Servier, licensed under a Creative Commons Attribution 3.0 unported license.

Figure 6 summarizes the main features of the current model of the axis ANGPTL3, 4 and 8 on selective tissue regulation of LPL following elegant proposals by Zhang [26,47,63]. 

ANGPTL3 is produced and secreted by the liver at a reasonably constant level all day. 

Since ANGPTL8 is lower during fasting (left column in Figure 6), there is a significant decrease in the levels of the active LPL inhibitor, the ANGPTL3/8 complex. Furthermore, apoAV and CREBH, which inactivate the ANGPTL3/8 complex, are produced in greater amounts in the liver during fasting. Consequently, all inhibition of muscle LPL is removed, and VLDL provides FA to oxidative tissues. Fasting concurrently causes a significant rise in ANGPTL4 synthesis (and a decrease in ANGPTL8), which in turn causes ANGPTL4 to locally block adipose LPL, hindering VLDL hydrolysis and diverting VLDL to oxidative tissues. Potentially futile cycles are thus avoided.

The levels of the active LPL inhibitor, ANGPTL3/8 complex, are elevated in the fed state (right column) due to a significant rise in hepatic ANGPTL8. This 3:1 ANGPTL3/8 complex functions as an endocrine inhibitor of LPL in oxidative tissues. Furthermore, eating decreases ANGPTL4 expression while raising ANGPTL8 expression in adipose tissue. Since the ANGPTL4/8 complex does not inhibit LPL, all inhibition of adipose LPL is removed, allowing adipocytes to be driven by high insulin levels to store fat appropriately by receiving it from VLDL and chylomicrons. Additionally, feeding significantly raises the amount of ANGPTL8 in WAT, where it combines with ANGPTL4 to create a complex (protein ratio 1:1). Although ANGPTL4 is a potent inhibitor of LPL activity on its own, ANGPTL8 lessens this inhibitory effect on LPL by creating the ANGPTL4/8 complex. Furthermore, WAT ANGPTL8 decreases adipocytes’ release of ANGPTL4. Thus, ANGPTL8 in WAT promotes LPL activity by inhibiting ANGPTL4 ‘s effect on LPL and ANGPTL4 adipocyte secretion. 

Thus, ANGPTL8 acts as a molecular switch that, in LPL inhibition, turns on ANGPTL3 but turns off ANGPTL4, balancing TG flow in various nutritional conditions. In the postprandial phase, why is the ANGPTL3/8 endocrine complex not acting on the adipose tissue, nullifying this process? 

There are several reasons, recently uncovered in elegant experiments by the Zhang group [26,47,63]:

- Firstly, ANGPTL8 breaks down ANGPTL4 and inhibits its secretion, and it also prevents ANGPTL4 from inhibiting LPL. After forming a strong bond with LPL, ANGPTL4 /8 is translocated to the capillary lumen;
- Secondly, plasminogen together with tPA binds to ANGPTL4 /8, which in turn produces plasmin. Plasmin then locally cleaves LPL inhibitors such as ANGPTL3 /8, ANGPTL4, and APOCIII. WAT LPL activity is thus entirely restored while the ANGPTL3 /8 is in turn capable of blocking oxidative-tissue LPL. The conversion of plasminogen to plasmin is made possible by the ANGPTL4 /8-LPL complex’s interactions with luminal plasminogen receptors and endothelial-released tPA;
- Plasma ANGPTL3/8 levels in healthy individuals are around 15 ng/mL while fasting but increase to approximately 28 ng/mL two hours after a meal. Consequently, fasting lowers the amount of ANGPTL3/8 in circulation, but considerable amounts are still present. This begs the following question: when fasting, how can the body restore oxidative-tissue LPL activity and dampen plasma ANGPTL3/8? The discovery that ApoAV is an endogenous ANGPTL3/8 inhibitor provides a first hint. By acting on the ANGPTL3/8 complex, ApoAV lifts LPL inhibition as shown earlier. It appears that the long-sought method by which ApoAV reduces serum TG is by selectively suppressing the ANGPTL3/8 complex’s LPL-inhibitory activity. The peculiar characteristics of this system contribute to the explanation of why, even after ApoAV was identified as a crucial component of TG metabolism two decades ago, the precise mechanism by which it reduces TG remained a persistent mystery [44,60,71,72].

## 9. ANGPTL8: Master Switch Gone Awry? 

LPL’s importance to the body’s overall energy metabolism has sparked a debate about its role in evolution [26,35,47]. 

Malnutrition was a major risk factor for survival during the evolution of humans. Evolutionary selection favored genes and genetic variants that lead to the accumulation of adipose storage, according to the thrifty gene theory, which holds that people with these genes survived famines by gaining more fat [26].

One may argue that ANGPTL8 is one of the genes because it is primarily involved in promoting fat storage following a meal. The same ANGPTL8 protein that most likely prevented hunger in the ancestors of humans now predisposes people to metabolic syndrome. Constant feeding increases circulating ANGPTL8 levels, which leads to increased adipose storage (obesity) and hypertriglyceridemia. 

From the foregoing, it appears that suppressing ANGPTL8 may be able to reverse thrifty features such as obesity, high TG levels, and metabolic syndrome. Obesity and serum TG levels are decreased in mice with ANGPTL8 deficiency. Adiposity and circulating TG levels were consistently reduced in mice given an ANGPTL8 Ab injection. It remains to be seen if this novel strategy can effectively reduce this element of metabolic syndrome in humans [47].

## 10. A Special Case: Postprandial Exercise

The flexibility of the ANGPTL regulation of fast and fed cycles is such that some apparent “exceptions” have recently come to light. Indeed, in the postprandial state, exercise selectively increased TG uptake and LPL activity in the skeletal muscle and heart by decreasing the ANGPTL3 activity [70].

Exercise, on a mechanistic level, reduced insulin secretion and reduced the transcription of ANGPTL8 in the liver. This transcription is necessary for ANGPTL3’s action via the ANGPTL3/8 complex. 

On the other hand, exercise increased the expression of ANGPTL4 in adipose tissues, overriding ANGPTL8/ANGPTL3’s regulatory functions and redirecting circulating TG away from storage. This is a previously unrecognized bifurcated ANGPTL–LPL network that overrides and coordinates fuel switching during aerobic activity [70].

## 11. Exchange of Lipids and Proteins Is Also a Key Component of the Intravascular Traffic of TRL 

Let us take a broad overview of the other key intravascular processes involved in the metabolism of chylomicrons and VLDL as we visit recent discoveries in the structure–function of LPL. The reader is directed to pertinent reviews for a thorough examination of these topics [6,7,9,21,37,38,73].

**Figure 7:**
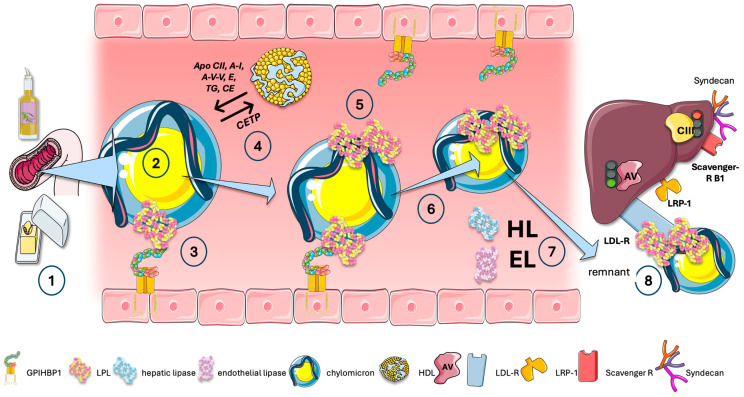
**Interaction of LPL with TRL and HDL, active forms.** We represent chylomicrons here; a similar process occurs in VLDL. (1) Intestinal chylomicrons, after passage through lymph (2), reach tissue capillaries which express LPL anchored by GPIHBP1 on the luminal surface of the endothelial cells and which supports the configuration of LPL to an active lipolytic enzyme (3). Important interactions with HDL occur all along the hydrolytic process: exchange activators such as apoCII and apoAV and inhibitors such as apoCIII, cholesterol, and phospholipids, and the action of CETP are key (4). Note that, in parallel, some LPL molecules can travel directly on the TRL particle, where it keeps its activity as it achieves optimal interface (5) and the particles are reduced in size by loss of TG to tissues and surface PL, etc. to HDL (6). Remnants are acted upon by hepatic lipase (HL) and endothelial lipase (EL) (7) to be finally taken up by several hepatic receptors including the LDL-receptor (LDL-R). Uptake is facilitated by the presence of LPL in the particles (8). LRP-1: LDL-like receptor 1; SRB1: scavenger receptor B1. The figure was partly generated using Servier Medical Art, provided by Servier, licensed under a Creative Commons Attribution 3.0 unported license.

In Figure 7, we represent CM for the sake of clarity; a similar initial process occurs for liver VLDL. The acquisition of exchangeable apolipoproteins (apos) from HDL happens in tandem with the liver and the intestine release of VLDL and CM particles into plasma. These surface proteins include apoAV and apoCII, which activate LPL, and other apos, most notably apoCIII and apoCI, which inhibit it. Additional proteins that are derived from HDL include apoE, apoAI, and apoAII.

Following the onset of lipolysis, as illustrated in Figure 7, exchangeable lipids are released into LDL and HDL, respectively. Cholesteryl ester transfer protein (CETP) facilitates the exchange of surface phospholipids and core lipids between TRL, LDL, and HDL. Since CETP transfers TG to HDL and cholesterol to the remnant or LDL particle, it is precisely this process that explains the frequent correlation between hypertriglyceridemia and low HDL cholesterol levels. This is the reason that low HDL cholesterol is not inherently harmful, but rather a sign of TRL dyslipidemia. It is a stand-in for a sign indicating atherogenic residues are present in the bloodstream. The importance of HDL in these processes cannot be overstated. The reader is referred to reviews on the matter [74,75,76,77].

Observe that some LPL molecules can move directly onto the TRL particle, where they optimize their contact to maintain their activity. TRL thus can carry enzyme and substrate concurrently [23,24,28,31].

These processes work together to create particles known as remnants, of which apoB48 and apoB100—the major structural proteins—are maintained in chylomicron and VLDL remnants.

During their passage through the hepatic sinusoids, remnant particles are further hydrolyzed by hepatic triglyceride lipase (HL) and acquire additional apoE. This, together with their attached LPL, aids in their binding and uptake by liver cell surface proteins, as illustrated in the figure. Furthermore, their apoAV content promotes further receptor-mediated remnant uptake [71,72]. Note that the series of reactions that stem from VLDL transformation into IDL and LDL are beyond the scope of this paper. Briefly, in addition to influencing LDL particle counts, elevated TRL and TRL remnant levels also significantly impact LDL particle size and composition and are associated with increased levels of small-dense LDL. Triglyceride-rich LDL is produced by increased VLDL production, ineffective lipolysis of TRL triglycerides, and/or decreased hepatic uptake of TRL remnants. The entire LDL size distribution will move toward smaller particles, small-dense LDL, which are more atherogenic than the bulk of LDL [9,15,16,17,18,21,37,78]. 

Recent elegant radioisotope kinetic research in humans has demonstrated a daily flux of 50 chylomicron plasma pools. These are significantly slower for VLDL fluxes, which are 10 pools for VLDL 1, 3.8 pools for VLDL 2, and 2 pools for IDL each day. Under normal metabolic settings, the reduced surface area of apoB48 compared to apoB100 and the higher size of chylomicron remnants make the binding of more apoE feasible. In consequence, they have a shorter plasma residence period (hours for VLDL versus minutes for CM) and higher hepatic uptake [9,38].

## 12. Blocking ANGPTLs: The Next Frontier?

Drugs that target LPL regulators, including ANGPTL3, ANGPTL4, ANGPTL8, ANGPTL3 /8, ApoAV, ApoCIII, and ApoCII, are being developed to achieve this goal [79,80,81,82,83,84]. These medications tackle different facets of TG metabolism and include mimetic peptides, antisense oligonucleotides, monoclonal antibodies, and small interfering RNAs [1,45,81,82,83,85,86]. It is critical to consider these emerging agents as complementary therapies that each target distinct aspects of TG metabolism rather than as substitutes for one another. A comprehensive approach holds promise for improving the management of metabolic syndrome and related conditions as our understanding of TG metabolism grows and as a wider range of LPL regulator-based drugs become more widely available. Consequently, we anticipate a higher degree of precision in the clinical regulation of TG metabolism [81,82,83].

**Figure 8:**
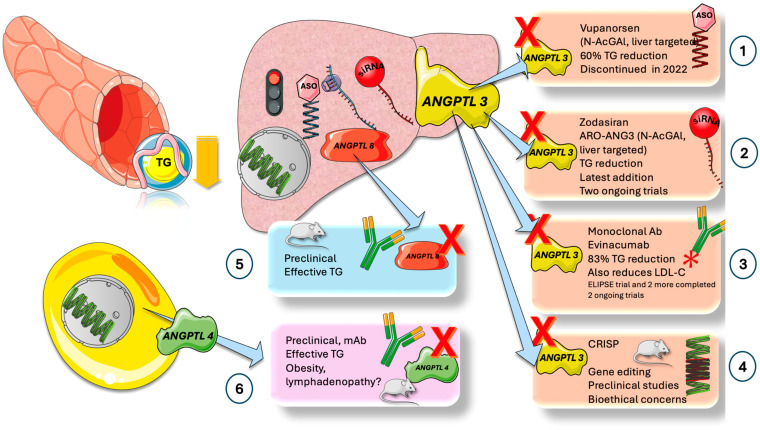
**The potential role of drugs targeting the ANGPTL3,4,8 axis in the treatment of TRL dyslipidemia.** In the past few years, elegant research on animals and loss of function studies in humans has earmarked both apoCIII and ANGPTL3 as pharmacological targets. Several trials are ongoing. ANGPTL3: Three approaches have been developed for the inhibition of ANGPTL3, ASO, siRNA, and mab. (1) Vupanorsen is an N-AcGal liver-targeted ASO of great effect but discontinued in late 2022. (2) Zodasiran is an ARO-ANG3, a N-AcGal liver-targeted siRNA and the latest addition to the armamentarium with two ongoing trials and (3) Evinacumab is a promising monoclonal antibody that provides up to 83% reduction in triglycerides and reduces LDL cholesterol. Elipse and two trials were completed, and two ongoing trials are taking place. Gene editing is feasible, and CRISPR has been conducted in animals but has obvious ethical issues (4). ANGPTL8: Given its pivotal role as a potential gatekeeper, intense research is being conducted at the preclinical level, so far (5). ANGPTL4 mAb is effective in TG reduction, though preclinical phase, obesity, and lymphadenopathy are concerns (6). The figure was partly generated using Servier Medical Art, provided by Servier, licensed under a Creative Commons Attribution 3.0 unported license.

Both apoCIII and ANGPTL3 have been identified as potential pharmaceutical targets in the last several years by elegant animal research and loss-of-function investigations in humans. In Figure 8, we summarize the current data on ANGPTL inhibitors.

*ANGPTL3:* ASO, siRNA, mab, and other methods have been developed for ANGPTL3 suppression. Vupanorsen was a very effective N-AcGal liver-targeted ASO; the trial, however, was stopped in late 2022. With two current trials, Zodasiran, an N-AcGal liver-targeted siRNA, and an ARO-ANG3 are the newest tools in the toolbox [87,88,89]. 

ANGPTL3 is bound and inhibited by the human monoclonal antibody Evinacumab [80,81,82,85,86]. Increased activity of endothelial lipase (EL) and lipoprotein lipase (LPL) is caused by this inhibition of ANGPTL3. EL is a phospholipase that reduces the amount of serum LDL-C while also selectively hydrolyzing high-density lipoprotein (HDL) phospholipids. Because of elevated LPL and EL activity TG, LDL-C, HDL-cholesterol, and VLDL-C are all decreased. The phase 3 ELIPSE trial was carried out following the determination of evinacumab’s proof of concept in a single-group phase 2 trial [81]. Evinacumab or placebo was administered to 65 individuals with homozygous familial hypercholesterolemia (HoFH) every 4 weeks. Without a substantial increase in adverse events, evinacumab decreased LDL-C by approximately 49% when compared to a placebo. It also markedly decreased non-HDL-C, total cholesterol, and TG by 50% on average, as well as ApoCIII by 90%, and ApoB100 by 37%. A second phase 2 trial was conducted on 272 patients who had refractory hypercholesterolemia. Evinacumab decreased LDLC levels by approximately 56%. Evinacumab inhibits ANGPTL3/8 more potently than it inhibits ANGPTL3 [56]. Evinacumab has been approved by the FDA and EMA to reduce LDL-C in patients with HoFH 12 years of age and older [81].

Though there are clear ethical concerns, gene editing is possible, and CRISPR has been tested on animals [47,79,81,82]. 

*ANGPTL8:* A great deal of preclinical research has been carried out thus far on this protein because of its crucial role as a possible gatekeeper, as stated above. Even with modest statin treatment, people with A8 truncating mutations frequently have low TG levels and high HDL cholesterol levels, which lower the incidence of heart disease. Therefore, ANGPTL8 inhibition could potentially boost HDL cholesterol and decrease TG at the same time [35,36,57,68,90].

*ANGPTL4:* A thorough grasp of its mechanism of action and the overwhelming genetic data support ANGPTL4 as a very desirable pharmaceutical target for decreasing plasma TG. However, the discovery that whole-body ANGPTL4 inactivation might cause serious clinical issues in mice has impeded the development of anti-ANGPTL4 methods. Mice lacking ANGPTL4 fed a diet high in saturated fatty acids have intestinal fibrosis, chylous ascites, fibrinopurulent peritonitis, weight loss, mesenteric lymphadenopathy, and eventual death. Mice injected with anti-ANGPTL4 antibodies showed similar pathological alterations but were successful in reducing TG in the preclinical phase [47]. 

## 13. Perspectives and Questions to Be Addressed in Future Research

**Figure 9:**
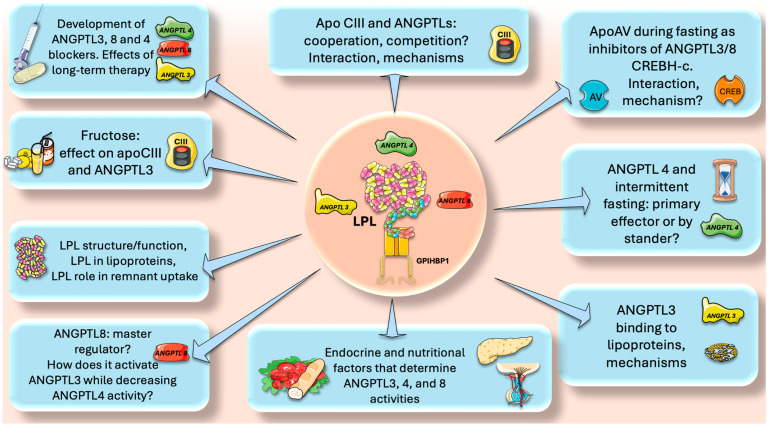
**Future directions for ANGPTL3, 4, and 8 research.** The figure was partly generated using Servier Medical Art, provided by Servier, licensed under a Creative Commons Attribution 3.0 unported license.

Herein, we summarized and put forward the concept that ANGPTLs and apolipoproteins are essential LPL regulators that have a significant impact on LPL activity. In this regard, we believe that, among other areas, research should focus on specific areas that we summarize in Figure 9.

1. Intermittent fasting and ANGPTL4. Multiple studies have demonstrated that intermittent fasting reduces insulin resistance, favorably shifts leptin and adiponectin levels, and is beneficial for weight loss [43,91,92,93,94,95,96]. Moreover, numerous disorders, such as obesity, type 2 diabetes, hypertension, and cardiovascular risk factors, can benefit from intermittent fasting, as shown by pre-clinical and clinical investigations [94,95,96]. As we have seen, ANGPTL4 is a master regulator of lipid traffic in the fasting state, shunting FA to muscle and reducing fat mass. How does intermittent fasting affect ANGPTL4? Is ANGPTL4 the main responsible for the lipid-lowering effects of intermittent fasting or just a bystander? Are there unknown regulatory loops ANGPTL4-adiponectin or ANGPTL4-leptin?
2. Endocrine and nutritional factors. The role of insulin in determining the correct expression of the LPL gene is well known. What are the roles of insulin and glucagon ratios in the expression of ANGPTL3,4 and 8? What are the other endocrine factors involved? Do incretins play a role? Regarding nutritional factors, fructose has received a lot of attention in the past decade as an important dyslipidemia CVD risk factor [97,98,99,100,101]. Indeed, our studies have shown (among other beneficial effects) that isocaloric fructose restriction reduced the AUC of TG, apoCIII, ANGPTL3, and apoB48, showing a continuous benefit throughout most of the postprandial period [20,73,102,103,104,105,106]. This has been corroborated by other studies that suggest that fructose-induced delayed catabolism of TRL is even more relevant than increased production [107,108]. Does fructose directly enhance ANGPTL3 expression?
3. ApoCIII and ANGPTLs, cooperation, competition? The role of apoCIII as a baseline inhibitor of LPL and its potential benefit throughout evolution has been discussed previously. One clear clue is the rarity of LOF mutations for apoCIII. What was protective in times of food uncertainty has become deleterious in times of caloric abundance. We have seen that on top of the apoCII/CIII regulation, there are the ANGPTL3,4, 8 axes. How do they interact? Do they compete? What are the mechanisms for putative additive or subtractive activities?
4. ApoAV and CHREBH-C activate LPL during fasting by inhibiting ANGPTL3/8. What are the precise mechanisms?
5. Plasmin clears up remaining ANGPTL3/8 complexes locally where ANGPTL4 is up (adipose capillary bed during fasting). What is the interaction between this process and the tPA-fibrinolysis pathway?
6. ANGPTL8 is a master switch in the fast-fed cycle. How does it, on one hand, activate ANGPTL3 whereas, on the other, decrease ANGPTL4 activity?
7. LPL structure-function. Much is known about this enzyme, but a lot remains to be uncovered, including its interaction with lipoproteins and its quantitative role in remnant uptake;
8. Development of new, effective, and safe ANGPTL blockers. ANGPTL3 inhibitors are already on the market, and new ones are continuously being developed. ANGPTL8 blockers show promise and ANGPTL4 blockers need more study. For all, what would be the effect of long-term therapy?

## 14. Conclusions

LPL’s critical function in TG trafficking and partitioning is now evident. When feeding, LPL activity rises in adipose tissue but falls in muscles; conversely, when fasting, LPL activity falls in adipose but rises in muscles. Although great progress has been achieved in the past few years, much remains uncertain regarding the mechanism controlling tissue-specific LPL activity during the fed-fast cycle. Medications that target LPL regulators—such as ANGPTL3, ANGPTL4, ANGPTL8, ANGPTL3 /8, and apo-AV, CIII, and CII—are being developed. These drugs, which include mimetic peptides, antisense oligonucleotides, monoclonal antibodies, and small interfering RNAs, target various aspects of TG metabolism. Furthermore, as ANGPTL8 functions as a switch, inhibiting it may be able to reverse thrifty traits including hypertriglyceridemia, metabolic syndrome, obesity, and CVD. In-depth studies in the upcoming years will undoubtedly illuminate this and several other unresolved issues.
